# “We might be making mistakes, but we’re learning, we’re trying, and we’re listening”: embedding qualitative methods within the plan-do-study-act cycle to support the development of a pilot multidisciplinary ARFID pathway

**DOI:** 10.1186/s40337-026-01692-w

**Published:** 2026-07-16

**Authors:** Maxine Byrne, Eva Eastman, Cat Papastavrou Brooks, Annie Stevenson, Natalie Wheelhouse, Sophie Webber, Lisa Bevis, Amy Brown

**Affiliations:** 1 SPIRED Research Clinic, Sussex Partnership NHS Foundation Trust, Mill View Hospital, Brighton and Hove, Hove, BN3 7HZ UK; 2 Washington Singer Laboratories, School of Psychology, University of Exeter, Perry Rd, Exeter, EX4 4QG UK; 3 Population Health Sciences, Bristol Medical School, University of Bristol, Canynge Hall, Bristol, BS8 2PN UK; 4 School of Psychology, University of Surrey, Elizabeth Fry Building, Guildford, GU2 7XH Surrey UK; 5 Norwich Medical School, University of East Anglia, Norwich, Norfolk, NR4 7TJ UK

**Keywords:** ARFID, Avoidant restrictive food intake disorder, ARFID pathway, MDT, Pathway evaluation, Eating disorder service, Adults

## Abstract

**Background:**

Avoidant restrictive food intake disorder (ARFID) was introduced to the Diagnostic and Statistical Manual of Mental Disorders (DSM-5) in 2013 and current guidelines recommend multidisciplinary care, however there are few evaluations of such pathways. This study aims to enhance the study stage of the plan-do-study-act (PDSA) cycle using qualitative methods to improve the development, refinement, and implementation of a novel pilot ARFID pathway in a community eating disorder service.

**Methods:**

Semi-structured interviews were conducted with 17 participants with experience of the pathway (7 service users:10 staff members) according to an interview schedule. Reflexive, codebook thematic analysis was used to analyse the interview data.

**Results:**

Gaps in understanding and treating ARFID were revealed, leading to feelings of uncertainty and frustration. The establishment of a specialist multidisciplinary team (MDT) improved knowledge and efficiency but created challenges related to team integration and resource allocation. Barriers for service user engagement and communication demonstrated the need for service user consultation and pathway co-creation.

**Conclusions:**

Service user consultation, treatment adaptations for neurodiversity and cross-service working were identified to improve the pathway as part of the act stage of the PDSA cycle. Future ARFID pathways should prioritise service user agency and access to multidisciplinary care and incorporate consultation from external eating disorder service, as well as lived experience experts.

## Abstract (plain language summary)

People who have avoidant restrictive food intake disorder (ARFID) have very limited diets and struggle to eat enough or certain kinds of food. This can cause problems with their health and daily life. A new pilot pathway in a community eating disorder service was set up to help adults with these eating difficulties.

To help improve this pathway we asked patients and staff what they thought about it. Seven patients and ten staff members were interviewed, and we organized what they said into key messages.

Both staff and patients said they were often unsure about what might be most helpful for people with ARFID. Having a team of different professionals made things easier, but it also brought some challenges. Patients said it was important to be involved in planning their care.

The study shows that care works best when patients are listened to, teams work together, and support is flexible to fit each person’s needs.

## Introduction

### Empirical background

Avoidant restrictive food intake disorder (ARFID) was introduced to the Diagnostic and Statistical Manual of Mental Disorders, 5th Edition (DSM-V) in 2013 as a Feeding and Eating Disorder, followed in 2019 by the International Classification of Diseases, 11th Edition (ICD-11). ARFID is characterised by the avoidance or restriction of food intake preventing nutritional and/or total energy requirements from being met, which is not motivated by preoccupation of body weight/shape. Consequently, individuals might experience significant weight loss, nutritional deficiency or dependence on enteral feeding or oral supplements, as well as interference with psychosocial functioning [1]. Within ARFID criteria, restricted food intake is not associated with weight or shape disturbance.

Consequences of food avoidance and restriction vary in severity. When compared to presentations of Anorexia Nervosa (AN), in clinical populations ARFID has been associated with earlier onset of eating disorder behaviours [2], prolonged duration of illness [3] and chronic, as opposed to acute weight loss [4]. However, ARFID’s clinical prevalence remains uncertain, with Van Burren et al.’s [5] recent review finding estimated prevalence rates to be between 0.3 and 15.5% in community-based clinical settings.

Despite this uncertainty, current findings offer encouraging evidence into the effectiveness of treatment approaches in reducing symptoms across adolescents and adults with ARFID. Emerging treatments include psychological interventions in inpatient [6], outpatient [7–9] and day-treatment settings [10, 11], in addition to nutritional rehabilitation [12] and psychotropic medication [13, 14].

Unlike other eating disorders, individuals with ARFID display significantly heterogeneous drivers for avoidant and restrictive eating [15], and the requirements of patients differ considerably depending on these drivers and symptomatology. As such, current UK guidelines for individuals with ARFID recommend assessment and treatment from a multidisciplinary team (MDT), including access to a variety of medical, psychological, dietetic, occupational, speech and language specialists [16]. Whilst there have been promising developments in treatment research, evidence relies largely on findings from single-intervention case series, chart reviews, case studies, proof of concept treatment studies and non-randomised clinical trials. As such, there are currently limited empirically supported, multidisciplinary treatments for adults with ARFID, and the National Institute for Health and Care Excellence [16] guidelines for eating disorder services contain no recommendations for ARFID treatment. Furthermore, whilst ARFID treatment research has greatly advanced, there is a lack of clarity around the applicability of these interventions within UK eating disorder services and there are significant knowledge gaps around how to best meet the needs of this complex, heterogeneous population in practice.

A recent review reported that adult community (outpatient) eating disorder services across the UK were not able to meet the National Health Service (NHS) England standards of providing timely and evidence-based ARFID treatment [17]. In response, an adult community NHS eating disorders service developed a pilot ARFID pathway to trial a multidisciplinary approach aimed at addressing the complex needs of individuals with ARFID. This paper aims to explore clinician and patient experiences of the pilot pathway to inform its ongoing development and refinement.

### Methodological background

Grant-funded, programme-level health intervention research typically uses qualitative methods at two key stages. Firstly to contribute to the intervention development process, and secondly to evaluate interventions when they have been implemented [18]. Embedding qualitative process evaluations within studies of complex interventions is particularly important in moving beyond effectiveness to assessing other impacts, relative value, mechanisms of action and how the intervention interacts with real-world context and systems [19, 20]. Qualitative research also has an important role in ensuring translation of health research into clinical practice [21].

This focus on embedded qualitative research as a key part of development and evaluation contrasts with how service development occurs in ‘real-world’ clinical contexts. Within resource-constrained mental health services, service development is often driven by policy and service pressures, progressing through staged governance gates: working group appointment, business case, pilot, implementation planning, and approval by senior management and commissioners [22]. Staff and stakeholder engagement does regularly inform service development, but it is rare to collect and analyse qualitative data from stakeholders [22].

There is a shift within health service research and development from summative evaluations to more iterative approaches better suited to continued learning and responsiveness within complex systems [23]. The PDSA approach offers a systematic iterative approach to health service development. It comprises four stages: *Plan*, which defines a goal and outlines service changes to achieve it; *Do*, which involves implementing the planned changes; *Study*, which evaluates the outcomes of the implementation; and *Act*, which uses these findings to refine and improve implementation. These four stages are repeated iteratively through cycles [24]. A systematic review of the use of the PDSA method to improve quality in healthcare [25], described 13 of the 73 included studies as using qualitative approaches at the *Study* stage. However, in fact only two of these studies collected data; e.g. either recording or taking detailed notes of interviews or focus groups to enable themes to be drawn from them. Neither of these used a formal qualitative method to do so. One stated that semi-structured interview data were used to “identify common themes” from reported experience [26], and one which used ranking of the most frequent responses and categorized these into “common meaningful themes” [27].

A previous study by this research group drew on elements of the PDSA approach [28] and utilized interim, integrated qualitative analysis of staff and patients as a way of getting preliminary feedback on a novel pathway prior to implementation. There has also been some qualitative work evaluating how staff engage with the PDSA cycle [29, 30]. However, to our knowledge there have not been any uses of formal qualitative methods integrated within a PDSA health service development study, despite repeated claims that the “flexible and qualitative nature” [31] of PDSA makes it suitable for engaging with the complex processes occurring in health care settings. This could be due to a lack of resources, knowledge, or skills within services to formally analyse qualitative data.

Collecting quantitative clinical or process outcomes in isolation makes it difficult to recommend specific changes which can feed into the *Act* stage, and given the natural variation in service outcomes, could be insufficient to recommend one PDSA cycle as superior. Where qualitative data is collected, but not formally analysed, insights could be missed, limiting the responsive potential of the PDSA process. This study aims to enhance the study stage of the PDSA cycle using qualitative methods to improve the development, refinement, and implementation of a novel pilot ARFID pathway in a community eating disorder service.

## Methods

This study explored clinician and service user experiences of a pilot multidisciplinary ARFID pathway, existing as a naturalistic service development within a resource limited NHS service. A qualitative design was utilized to enhance the study stage of the PDSA cycle, to improve the development, refinement, and implementation of a novel pilot ARFID pathway in a community eating disorder service.

### Pathway description

#### Pathway model

The ARFID pathway is a pilot, multi-disciplinary outpatient treatment pathway for individuals aged 18 + with ARFID, developed by and embedded within an NHS community adult eating disorder service. Pathway development and implementation relied on existing resources and did not receive any additional funding. A total cohort of 26 service users were accepted onto the ARFID pathway based on the following triage criteria: individuals receiving an ARFID diagnosis according to the DSM-5 criteria at assessment, physical and mental health risk currently managed in the community (i.e., not requiring inpatient medical admission), and willingness and capacity to engage in regular, outpatient multidisciplinary treatment. Due to service pressures of managing staffing resources with the high number of referrals received, in October 2022 - December 2023 an additional triage criterion: individuals with a Body Mass Index (BMI) of < 15, was implemented. No exclusions were made based on comorbidity, or previous treatment.

**Fig. 1:**
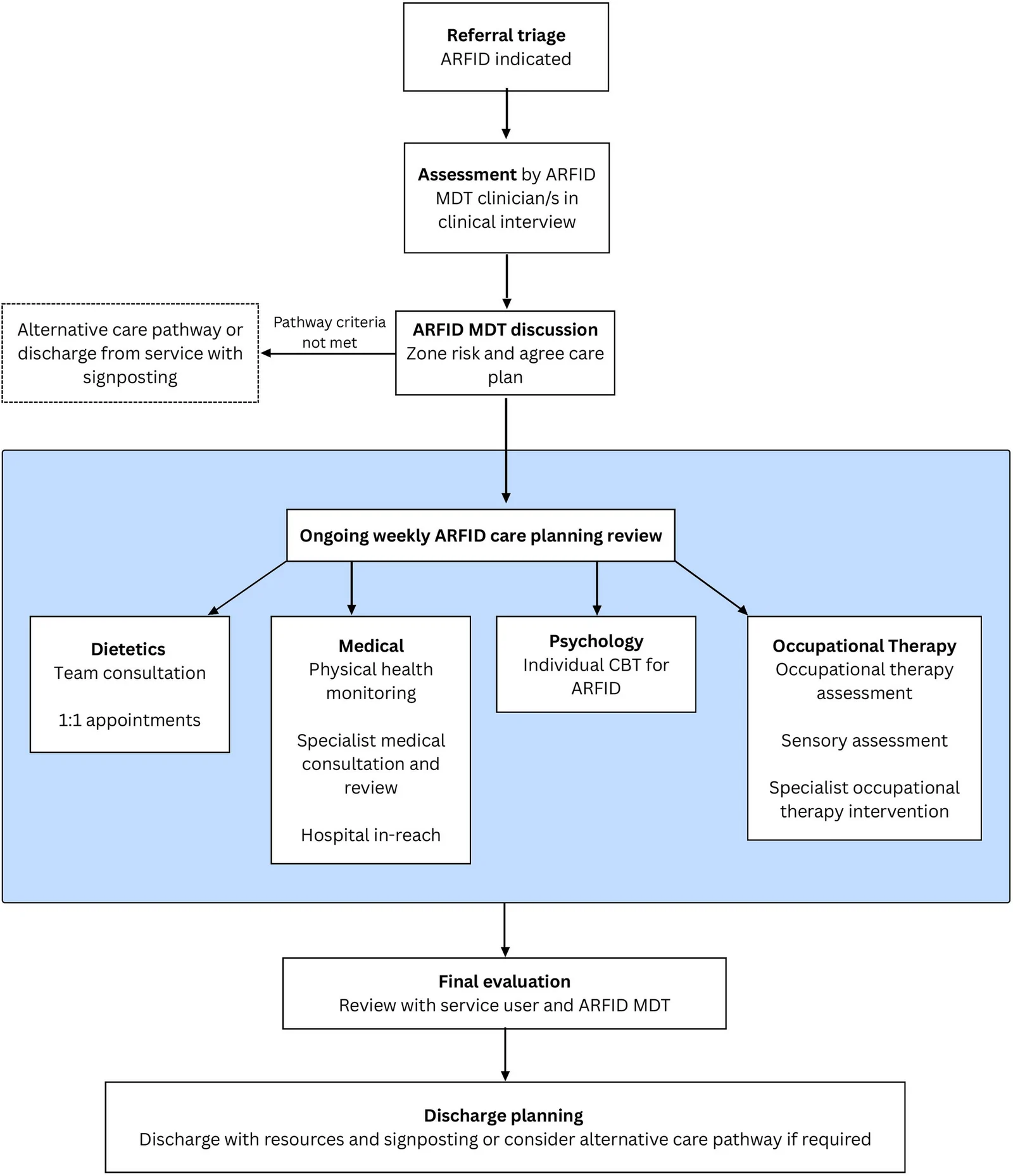
Pathway model

The pathway aimed to monitor individual’s physical health, weight restore, improve nutritional health, enhance quality of life, and/or reduce dependence on enteral feeding. Interventions included specialist medical interventions, psychological treatment (CBT-AR: [32] according to the manual), specialist dietetic management and specialist occupational therapy (OT) input. These were delivered by a multidisciplinary ‘mini-team’ of medical, psychology, dietetic and occupational therapy professionals, led by a staff ARFID Champion nominated to lead the pathway development, and were decided on a case-by-case basis, according to clinical need. As such, service users received an individualised care pathway, accessing interventions with members of the MDT as appropriate, according to the pathway model below. Pathway processes including eligibility criteria and care planning were made independently from the research team and were grounded in the demands of a NHS eating disorder service responsive to the challenges of delivering care within the existing, resource-limited system. See Fig. 1 for an overview of the pathway model.

#### Service user involvement in pathway development

As the pathway progressed and service users were accepted for treatment, feedback was requested via a service-specific, informal questionnaire issued at the end of their care pathway. This informed interim pathway development but was separate from this current research study.

#### Initial pilot evaluation

The current research followed on from an initial evaluation, conducted by the same NHS service [33] which was used to refine the current research project's interview schedule. Two service users were interviewed about their experience of the pathway and findings recommended the facilitation of an ARFID peer support group, provision of a nominated clinician to contact for support, inclusion of meal planning, and consideration of financial support during treatment. It was identified that future research could benefit from the inclusion of a larger sample, as well the perspectives of clinicians, which the current research aimed to achieve.

#### Service user involvement in research study

Service users were also invited to join the current evaluative project as lived experience co-researchers; one person with experience of the ARFID pathway joined the project in this role. They were involved in all aspects of the data analysis process, including coding transcripts and developing themes, and were supported in doing this work by the NHS trust’s patient and public involvement team. This support was particularly important as they were the only person working in a specifically lived experience role on this project, although many other team members also had lived experience of ARFID and neurodivergence.

### Participants

**Fig. 2:**
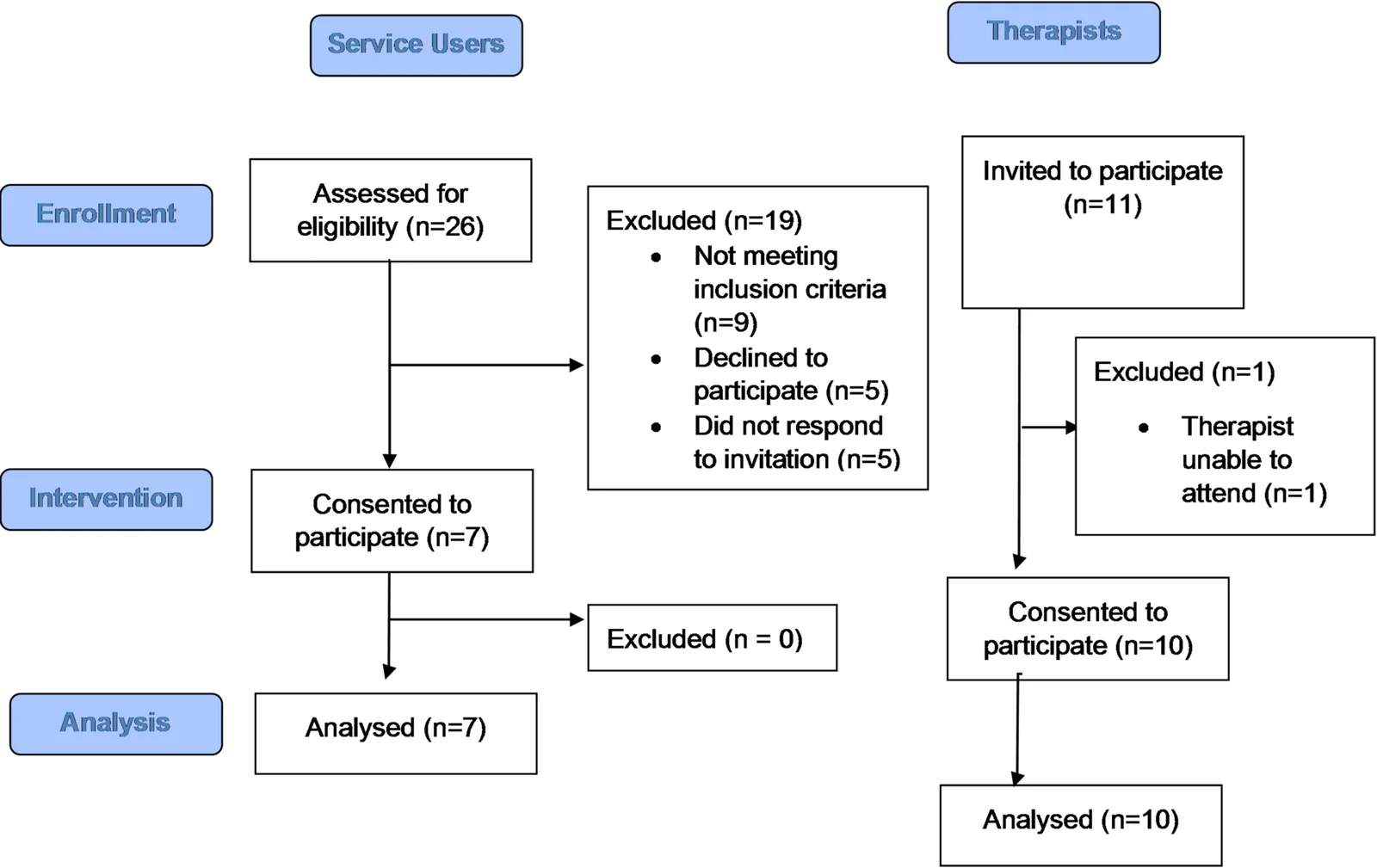
Consort

Inclusion criteria for participants for this research study included English speaking and having capacity to consent to research. In addition, service users were required to have attended a minimum of three appointments within the care package offered. Of the 26 service users accepted for the pilot ARFID pathway treatment, 15 were assessed as eligible for the research interview and seven provided consent. The final sample was also composed of 10 staff members, five of whom delivered ARFID pathway treatment and five who delivered other eating disorder interventions (e.g., for Anorexia Nervosa or Bulimia Nervosa). These non-ARFID pathway clinicians were selected to provide insight into the pathway delivery from the perspective of the wider MDT. The participant selection process is presented below in Fig. 2 and the participant demographics are presented in Table 1.

**Table 1:** Service user clinical characteristics

		Service users (*N* = 7)
Age (M ± SD)		21.14 ± 2.55
Body Mass Index (BMI)		16.84 ± 1.49
Treatment		
No of sessions		27.14 ± 11.38
Completion rate		89.14% ±15.36%
No of professions seen		2.14 ± 1.08

Because of the small sample size, we have not reported demographic data to avoid identifying participants. There was a roughly even split in terms of sex among service users, and all the eating disorder clinicians interviewed were female. Over half the service users were White British. Most patient participants had an onset of eating disorders that was greater or equal to 3 years. Most patient participants were autistic, and a minority had ADHD. There were a range of clinical roles sampled including occupational therapy, counselling psychology, clinical psychology, dietetics, psychological therapies, and assistant/trainee psychologists.

### Data collection

Demographic and intervention data were collected for every service user referred into the ARFID pilot from March 2021–August 2024, including those who later consented to interview. Demographic data included age at the time of referral, sex, neurodivergence and time since onset of ARFID. Demographic data was collected from referral records received by the service and/or clinical system records. Intervention data including number of appointments offered, appointments attended, and professions seen was collected from clinical system records.

Service users accepted onto the ARFID pathway within the time period specified and staff members working within the service were also invited to participate in a research interview. An open-ended interview schedule exploring experiences of the pilot ARFID pathway was created by the research team with consultation from a Lived Experience Expert, see Appendix 1. Interviews took place virtually and individually, conducted by MB, EE and SW and were audio-recorded and transcribed, lasting on average 42 min.

### Data analysis

The pathway evaluation aimed to identify patterns and commonalities in staff and service user experiences of the ARFID pilot pathway and employed a codebook thematic analysis to help interpret meaningful patterns [34]. Two researchers MB, EE and SW with access to service user data cleaned and anonymised the transcripts, replacing names with participant IDs and removing identifying information. Five researchers then familiarised themselves with the anonymised transcripts before taking an inductive coding approach to the data; meeting weekly to discuss and define any new codes generated and update the codebook. Three transcripts were double coded to ensure researchers held a shared understanding of the transcripts. The codebook was made available as a shared document to allow all researchers access and editing ability. Once all transcripts were coded and the codebook finalised, initial themes were developed by three of the researchers (MB, EE and CPB), using a mapping approach to synthesise codes into themes. These themes were reviewed, discussed with all authors and finally, defined.

### Reflexivity

As a research team we reflected on the role our positionalities brought to the data collection and analysis by engaging in regular reflexivity peer supervision. Three members of the team held or currently hold clinical positions on the ARFID pathway and contributed to its development. Reflexive discourse revealed the researchers’ perceived challenge in navigating these dual roles, particularly during interviewing and data analysis. In addition, several members of the team also have lived experience of ARFID, another eating disorder and neurodivergence. Whilst the researchers attempted to remain grounded in the participant’s voices, readers should be aware the intersectionality of these personal histories likely shaped our interpretive lenses.

### Ethics

The project was registered as a service evaluation on the 10th of August 2022, by the Quality Improvement Support team for the participating NHS trust, according to their trust guidelines. British Psychological Society (BPS) ethical guidelines [35] were followed by all researchers working on the project. Participants were provided with an information sheet informing them of the aims of the study and a consent form and were advised of their right to withdraw. Service user participants were also advised their interview would have no impact on the availability or delivery of future treatment within the service. After interviewing, participants were debriefed and signposted to further psychological support if necessary. All names have been replaced with participant numbers and any identifying information has been removed.

## Results

**Fig. 3:**
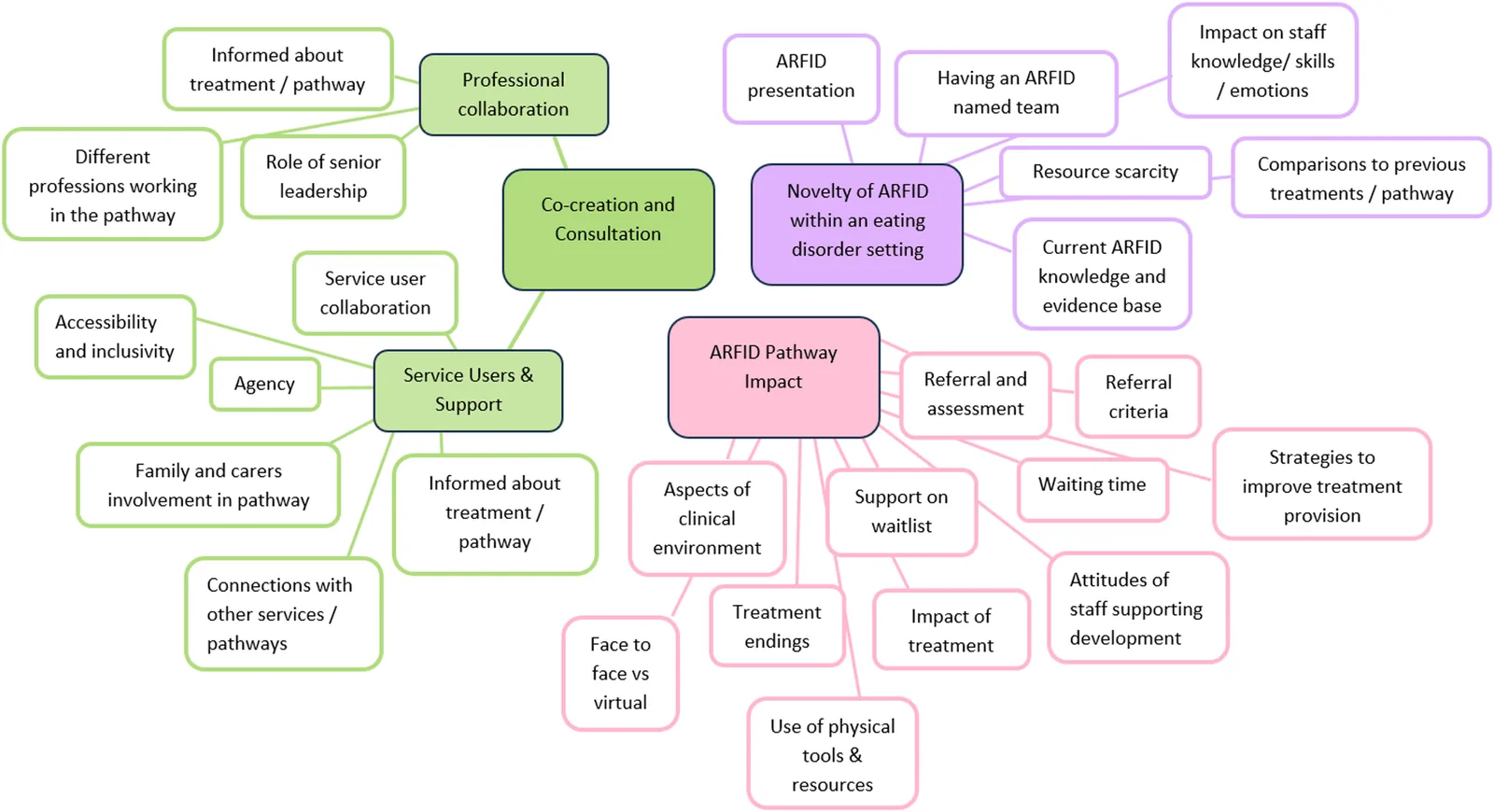
Thematic map

Three themes were developed during thematic analysis: Novelty of ARFID within an Eating Disorder Service (1), ARFID Pathway Impact (2) and Co-creation and Consultation (3). Themes and sub-themes are presented in a thematic map (Fig. 3).

### Theme 1: “A real sense of uncertainty”: novelty of ARFID within an eating disorder service

Participants emphasised the current state of ARFID knowledge as underdeveloped and not well understood. Consequently, for some service users their experience of ARFID during their lifetime had led to instances of feeling misunderstood by professionals, inappropriately treated, or even not treated at all.

*“I think for me because I was misdiagnosed… I was put into hospital and treated for the wrong thing. So then that caused a lot of*,* because I didn’t know what I had at that point*,* it caused a lot of trauma.” Service user*,* P05*

Other service users reported this lack of understanding about their eating disorder left them feeling isolated, confused, and experiencing *“distrust in the system” (Service user*,* P05).*

Staff members also highlighted the scarcity of research, knowledge and guidance surrounding ARFID, particularly in adolescent and adult populations.

*“It was a somewhat overlooked group*,* I would say*,* and a misunderstood group*,* a not well-researched group” Consultant clinical psychologist*,* P20*

This lack of guidance resulted in processes which were *“inconsistent and very confusing” (Occupational therapist*,* P15)*, and was perceived as a significant barrier to providing high quality care. For many, this resulted in feelings of uncertainty and frustration among clinicians, with one staff member pointing to the dearth of knowledge surrounding ARFID leaving many professionals feeling *“de-skilled” (Consultant clinical psychologist*,* P20).*

Lack of knowledge and expertise within the service was widely recognised by staff members and so the development of a specialist mini-team and named ARFID champion was felt to significantly increase team confidence and pathway clarity.

*“I think*,* for me*,* there was a really clear person that was the go- to*,* in terms of process and knowledge and information… it was clear that knowledge sat with them.” Clinical psychologist*,* P14*

This concentration of expertise also improved the efficiency of referrals and assessments and fostered a more supportive working environment where staff felt comfortable to *“ask questions or share thoughts”* about ARFID, *“share[ing] that burden of not really knowing what to do” (Psychological therapist*,* P18).*

Despite this, several staff members expressed concerns that the separateness of the mini team reduced team *“ownership”* and opportunities for professionals to develop ARFID specific clinical skills *(Consultant clinical psychologist*,* P20)*.

*“It definitely felt like*,* and continues to feel like*,* it’s very specialist and that there’s only a small number of clinicians that offer the expertise. I think it feels like a limitation.” Clinical psychologist*,* P14*

Most staff members also suggested the wider community’s growing awareness of ARFID could significantly impact the services capacity to respond effectively, given its current allocation of resources.

*“With the amount of staff resources that we have*,* we’re just going to see an increase in waiting lists*,* an increase in pressure and demands to treat these people*,* which I don’t think we have the resources for.” Psychological therapist*,* P18*

There was a shared belief among staff members that the current service model is not equipped to cope with the anticipated increase in referrals, with one participant describing it as feeling of *“balancing on a tightrope” (Psychological therapist*,* P18).*

### Theme 2: “We’re not friends, but we’re acquaintances, me and food”: ARFID pathway impact

Service users reported the pilot treatment led to significant and wide-ranging improvements, including developing greater self-awareness, learning practical skills and coping strategies, fostering social benefits and improved nutritional health and self-confidence.

*“It’s given me the skill set to tackle it myself as well and not get too ill. I’ve always had issues with eating*,* and I’ve still got them*,* but I can actually eat a more varied diet*,* and I can try different things.” Service user*,* P04*

Despite this, there were several aspects of treatment which negatively affected its perceived impact. While some service users felt *“looked after” (Service user*,* P04)* and reassured by physical health checks whilst waiting for treatment, most found the extended wait time challenging, forcing service users to manage their symptoms independently, leading to therapeutic ruptures later in treatment.

*“Whereas if I had had it back in when I first spoke to them*,* I think it was probably 2021 or something*,* I don’t know exactly. But whenever it was*,* whenever I first spoke to them*,* I was really bad and I was really underweight and really ill… So*,* it was a long time to deal with that on my own.” Service user*,* P10*

Virtual support was convenient and accessible for most participants, particularly for those who found it challenging to leave home. However, many also highlighted challenges associated with this form of treatment, some felt self-conscious, *“embarrassed to go on calls” (Service user*,* P10*) and others found virtual support limited practical skill development, application of learning or family inclusion.

*“I think sometimes online*,* it’s not always easy to really feel in it*,* because when you’re in your own home*,* you don’t feel like you’re out of that environment. And when you’re out of the environment*,* you can actually take that information and put it back into the environment.” Service user*,* P10*

The pathway’s (later introduced) BMI referral criteria sparked significant discussion among staff participants, with many feeling the criteria was *“at odds with what we value as a service” (Assistant psychologist*,* P16)* and inequitable compared to other pathways.

*“But it’s unfair that you have to be very poorly to get treatment. We don’t expect that from of any of our other patients.” Consultant clinical psychologist*,* P20*

Whilst there was a strong *“appetite for change” (Assistant psychologist*,* P16*) to lessen this criterion, resource constraints were highlighted by many as a major barrier.

*“I know initially I said the BMI cut off is a problem*,* I think it’d be even more worrying if we didn’t have one from a capacity perspective.” Occupational therapist*,* P15*

Accordingly, many staff advocated for a stepped care model, including improved signposting, low-intensity guided self-help or group work for those not meeting the criteria for individual treatment. Participants reflected these approaches might allow for increased treatment accessibility whilst balancing limited staff resources.

*“An ARFID group for people that meet criteria but don’t have such a high physical risk…that could be a good way of pooling the resource that we do have and be able to share that out*,* disseminate that out in a way that reaches quite a lot of people.” Clinical psychologist*,* P11*

Reflecting on the rapidly evolving landscape of ARFID knowledge and research, clinicians emphasised several improvements that could have better supported the pathway’s implementation. To support the pathway to feel more manageable, clinicians suggested trialling the pathway with a small, defined group of individuals.

*“So rather than just opening it up and that being your future*,* doing it with a small pool of people that’s contained*,* that you can then step back from*,* analyse the data*,* and then make a decision as to whether or not it’s feasible and acceptable to continue.” Psychological therapist*,* P18*

Other participants outlined procedures including defining outcome measures and creating questionnaire packs, naming MDT members involved, identifying and meeting training needs and establishing supervision structures, all prior to the pathway’s launch. These suggestions were felt to support in more effectively piloting the ARFID pathway, whilst also ensuring a clear endpoint and space for evaluation.

### Theme 3: “We need to understand and listen and act on their experiences”: co-creation and consultation

#### Subtheme 3a: service users and their support networks

Participants emphasised the importance of service users collaborating with clinicians to shape their treatment. Service users reported that having agency over their own care plan helped them to feel in control and autonomous, with one service user reflecting *“I chose what we do each week*,* what we tackle*,* what I wanted to talk about and what I needed help with” (Service user*,* P04).* The valuable role of service user feedback was also emphasised more widely, in pathway co-creation.

*“Perhaps within a year’s time*,* we will have another X amount of clients that completed treatment in the sense that we can learn from. And learn from their outcome measures*,* and the feedback that they’ve given us about the treatment that we’re offering.” Counselling psychologist*,* P19*

In addition, collaboratively exploring and implementing treatment adaptations, particularly for those with co-occurring conditions or neurodivergent traits, was also felt to increase service user accessibility and inclusivity for those individuals.

*“We can adapt it and individualise it for someone if they have a particular sensory need or nutritional deficiencies or something*,* that is embedded in the pathway” Clinical psychologist*,* P11*

However, several service users called for further development to support diverse communication needs and preferences. For several, long appointments felt *“overwhelming” (Service user P04)* and treatment courses did not factor in enough time to build a meaningful and safe therapeutic relationship.

*“Just because if you jump straight into food*,* you know*,* that’s the thing that my entire life has been about*,* is like*,* whoa. Like this is a bit harsh to me.” Service user*,* P05*

The importance of collaboration between service users and their chosen support networks, particularly families, was also raised, with one participant explaining *“my family don’t really get it*,* they don’t really understand what it is*,* they kind of just call me lazy” (Service user*,* P07)* illustrating the need for family education and support. For some staff participants, family involvement *“helped to unlock changes” (Assistant psychologist*,* P17)* service users hadn’t thought about, but for others family involvement was perceived as *“take[ing] away from the actual treatment” (Service user*,* P04).* Overall, this underscores the importance of a balanced approach to family involvement, where service users remain at the *“main core” (Service user*,* P05)* of the intervention.

#### Subtheme 3b: professional collaboration

Collaboration among MDT professionals within the pilot pathway was also highly valued in addressing ARFID in a holistic manner.

*“There is that really good*,* across-board relationship with the different modalities of care. It looks like we’re trying to look at some people’s journeys and piece that all together*,* the narrative.” Dietician*,* P13*

Many staff members reflected that this collaborative effort allowed them to deliver *“incredibly adaptive” care plans with a “massive ability to tailor to an individual[s] situation”* and needs *(Clinical psychologist*,* P14).* In particular, the Occupational Therapy role was highlighted for its value in assessing individuals’ sensory needs and *“supporting people in alternative ways to engage” (Occupational therapist*,* P12).*

Value in collaboration also extended beyond the service, to other teams delivering ARFID interventions. Participants emphasised the benefit of liaising with external services for consultation, training, and to share knowledge and experiences.

*“Learning from people that might be six months to a year ahead of where you’re at*,* and then you can learn from them*,* what’s been working about the pathway they set up and the treatments that they’re offering and what they may have done.” Counselling psychologist*,* P19*

Building and maintaining these connections not only facilitated learning but also fostered a culture of shared responsibility and innovation, with clinicians feeling encouraged to *“keep pushing it [the pathway] forward” (Consultant clinical psychologist*,* P20).*

## Discussion

The findings of this qualitative evaluation highlight both challenges and benefits of the pilot ARFID pathway from the perspectives of service users and MDT clinicians. Reflecting on the underdeveloped state of ARFID knowledge, clinicians reported that the pilot pathway unearthed feelings of uncertainty and confusion. Despite having evolved from the DSM-5’s diagnostic criteria for Feeding Disorder in Infancy or Early Childhood [1], ARFID is frequently referred to as a new eating disorder within the wider eating disorders community. Whilst this represents positive change, raising awareness and advancing developments in treatment, this conceptualisation risks overlooking the vast literature on paediatric feeding disorders, which hold important insights into effective interventions and patient characteristics [36]. Indeed, professions effectively addressing ARFID driven by feeding disturbances within paediatric services, such as Speech and Language or Neurodevelopmental specialists, remain poorly incorporated in adolescent and adult eating disorder services [32]. Collaborating on ARFID pathways and improving joined-up care may, therefore, bring significant opportunities for adolescent and adult eating disorder and paediatric feeding and eating disorder services to integrate their knowledge and enhance ARFID care provision.

Whilst more integrated care pathways between paediatric and adult services may be valuable for eating disorder services, recent research suggests unique differences in demographic findings, medical and psychiatric comorbidities and symptoms in paediatric and adult ARFID populations [37]. Furthermore, treatment outcomes varied in a trial of CBT-AR, with significant differences in treatment efficacy found between adult and paediatric populations [38], possibly reflecting these differing characteristics. Consequently, adolescent and adult care pathways for ARFID may require a departure from the well-established paediatric feeding interventions. Staff participants involved in the current evaluation emphasised that implementation was grounded in patient-centred discovery and continuous integration of patient feedback. Adult eating disorder services may therefore be best served by finding a middle ground, aligning research and care pathways and fostering closer collaboration with paediatric colleagues, whilst also distinctly consulting with adult populations in the development of ARFID pathways.

Staff participants reflected that incorporating service user consultation and co-creation during the pathway’s developmental stage might refine the pilot’s implementation. Reay et al. [39] exploring the service needs of a pathway for individuals with longstanding eating disorders, also found service user consultation was instrumental in defining pathway aims and identifying preferred treatment interventions and formats. Numerous policies and research support this approach, advocating for the active involvement of individuals with lived experience in the co-production and co-delivery of eating disorder services [40, 41].

However, co-developing ARFID pathways with lived experience experts may prove challenging, given the novelty of treatment for ARFID within eating disorder services. Adult eating disorder services might, therefore, benefit from collaborating with teams currently delivering ARFID pathways and engaging lived experience experts for cross-service consultation.

According to staff participants, team-wide appetite for change was crucial for pathway development and individuals were driven to provide equitable care. This finding aligns with Hyam’s [42] suggestion that the perceived value of an innovation is an important enabling factor for engagement and adherence to a novel pathway. It is clear from these findings that growth-orientated services and individuals are, therefore, crucial for successful pathway implementation.

Staff participants in the current evaluation reported that collaborative, MDT working facilitated holistic care, which particularly benefited neurodivergent individuals requiring pathway adaptations. A recent review highlighted the need for such adapted processes, indicating that the co-occurrence of ARFID in individuals who are autistic ranged from 8.2% up to 54.75% in clinical samples [43]. However, there are challenges in making these adaptations. Disentangling eating behaviours between ARFID pathology and autism, such as textural sensitivities or preference for predictability, can be complicated [44]. “An MDT approach is therefore likely to be the most effective way to assess and support individuals who are autistic with ARFID, whilst incorporating specialist autism services for adaptation recommendations. Further, within ED treatment, collaboratively working with an individual to respect their needs and provide neuro-affirming care and adaptations is recommended [45, 46].

Staff participants emphasised the role of Occupational Therapy (OT) in the ARFID pathway. OT expertise in assessing individuals’ sensory and occupational needs, delivering occupationally informed interventions and providing recommendations for adapted treatment is particularly relevant given the high prevalence of neurodivergence within the ARFID population. Using sensory, biomechanical and occupational performance frames of reference, OT approaches are well-positioned to assess and address the often complex interplay of psycho-social, occupational, sensory and motor factors that influence an individual’s well-being [47]. These interventions can complement psychological treatments, offering a more holistic approach to care. While psychological interventions appear to be the primary focus of current ARFID treatment research, the role and scope of OT approaches within an interdisciplinary pathway warrants further exploration.

Exploring individual barriers to treatment, service user participants reported that long session durations felt overwhelming, while short treatment lengths reduced their ability to build meaningful therapeutic relationships. These findings align with Kinnaird et al. [48], who interviewed service users who are autistic and have Anorexia Nervosa (AN), similarly highlighting how short treatment time-frames diminished treatment accessibility. These findings corroborate the value of adaptations according to individual needs, such as breaks in therapy, shorter session bursts and longer courses of treatment for individuals who are autistic [49, 50]. Notably, much of the existing treatment adaptation guidance for individuals who are autistic with an eating disorder is grounded in research of AN. Consequently, little is known about how to provide adaptations for those with co-occurring ARFID and autism [51]. Furthermore, the comparative efficacy of existing adult ARFID treatment interventions has also yet to be investigated in a clinical population who are Autistic. Given marked differences in eating disorder pathology between AN and ARFID, as well as variations in ARFID pathology [52] and health outcomes [51] between individuals who are autistic and those who aren’t, and need for further treatment into overlap and coexistence of ARFID and other eating disorders [53–56] more detailed treatment guidance and adaptations are needed to support this population.

### Strengths and limitations

A central strength of this study was that pathway development and implementation happened naturalistically in response to service needs, and researchers were embedded within the clinical service. This approach is aligned with Medical Research Council guidance on complex interventions, recommending research is used to support real world decision making [20]. A further strength of this evaluation was its rich understanding of the facilitators and barriers to the pathway implementation gained from both service users and staff members. Interviewing staff members external to the pathway may have facilitated a more balanced evaluation, minimising the likelihood of purely favourable statements from clinicians currently involved in its delivery.

One key limitation was the small service user sample size, however demographic characteristics indicated it was representative of a community-based clinical ARFID sample. In addition, to gain insight into the entirety of the pathway processes, service users who attended less than three scheduled appointments were excluded from the study. These voices, who likely have valuable insight into the barriers and challenges associated with accessing ARFID treatment, have therefore gone unheard. Similarly, due to resource constraints, service users’ support networks were also not interviewed, who may have provided further insight into the experience of family involvement. We did not collect data on gender identity as part of our participant demographics, which could be especially problematic for this clinical population as neurodivergent people are more likely to be trans or nonbinary [57] and trans and non-binary people also have higher rates of eating disorders [58]. There may be gender-neurodivergent-sensory intersections experienced by this subgroup [59].

### Clinical implications

For future service development, ARFID pathways should ensure service users’ ability to access a holistic, multidisciplinary care pathway. Interventions should be adapted to neurodiversity needs and service users offered peer support connections. Staff also underscored the value of cross-service collaboration, in addition to the active involvement of service users in pathway co-creation and ongoing consultation. Considering the number of service users who attended less than three scheduled appointments (*n* = 9), future research might also explore the experiences of those with reduced engagement to better understand its barriers. Clinicians working within the current pilot pathway will aim to implement these findings: improving treatment adaptations for neurodivergence, integrating third party service collaboration and service-user consultation into future developments and exploring opportunities for peer support. The service also aims to secure funding for an established ARFID pathway with increased resources, using these findings as support for its acceptability and feasibility.

## Conclusion

This study used thematic analysis to enhance the study stage of the plan-do-study-act (PDSA) cycle to improve the development, refinement, and implementation of a novel pilot ARFID pathway in a community eating disorder service. Most participants found the pilot pathway beneficial and reported significant improvements in physical health, mental health and overall quality of life. Participants identified exerting treatment agency and accessing, or working within an MDT providing holistic care were valuable characteristics of the pathway. Service user consultation on pathway implementation, cross-service collaboration and further adaptations for engagement and communication barriers were highlighted as suggestions for future improvements. This embedded evaluation provides initial support for the value of multidisciplinary, adult ARFID pathways within eating disorder services.

## Data Availability

Anonymised interview data can be provided upon request to the corresponding author.
